# Research status and future focus on *Codonopsis pilosula*: A bibliometric analysis of past and present studies

**DOI:** 10.1016/j.heliyon.2024.e40069

**Published:** 2024-11-05

**Authors:** Doudou Yang, Qingqing Zhang, Zhuodi Wu, Yuan Chen, Ziping Cai, Liang Zhao, Dandan Zhang, Yinku Liang

**Affiliations:** a Collaborative Innovation Center for Comprehensive Development of Biological Resources in Qinba Mountain Area of Southern Shaanxi, Qinba Provincial Key Laboratory of Biological Resources and Ecological Environment, Shaanxi Province Key Laboratory of Bio Resources, College of Biological Science and Engineering, Shaanxi University of Technology, Hanzhong, China; b College of Agronomy, Gansu Agricultural University, Lanzhou, China; c Institute of Chinese Herbal Medicine, Institute of Dryland Agriculture, Gansu Academy of Agricultural, Lanzhou, China; d Kangxian County Agricultural and Rural Bureau of Gansu Province, Longnan, China

**Keywords:** Herb, Knowledge map, Research progress, Frontier focus

## Abstract

**Background:**

*Codonopsis pilosula* is a traditional herb widely used in Asian countries. As regulatory institutions increasingly acknowledge its medicinal and food-related characteristics, the attention from scholars is expected to increase rapidly. However, an overview and reports on research focus on this promising herb are currently lacking. Therefore, the objective of this study was to fill this knowledge gap by conducting a systematic review of relevant literature to explore the developing history and research focus on *C. pilosula*. This research is of significant importance in gaining a deeper understanding of the potential benefits and application areas of *C. pilosula*.

**Method:**

The output, institutions, countries, authors, highly cited publications, frontier focus and funding agencies of *C. pilosula* were analyzed using VOSviewer, CiteSpace, and COOC software.

**Results:**

The development of *C. pilosula* experienced 3 stages: initial stage (1979–2002), fast stage (2003–2012), and explosive stage (2013-present), with each stage showing linear growth. Worldwide attention from 1292 institutions in 27 countries have been paid, with China leading in output and collaboration. Lanzhou University in China contributed most publications with Fangdi Hu as a core specialist facilitating collaborations in agricultural and pharmacological research. A stable cooperation network has formed among researchers from different fields, focusing on pharmacological activity, chemical constituents, quality control, and medication rules. The research focus updated timely with new scientific frontiers occur. Medication rules, data mining, molecular docking, traditional Chinese medicine, gut microbiota, and polysaccharide are frontier focus presently and in the near future. Significant attention and funding have been provided by the national and local government of Gansu province in China.

**Conclusion:**

Research on *C. pilosula* is thriving, particularly in China, with a promising future in traditional Chinese medicine and pharmacology. This study provides valuable insights for future research directions and contributes to the effective development of *C. pilosula*.

## 1 Introduction

Chinese herbal medicine has been attracting increasing global attention in the past decades, it has been existing in China for over 2000 years [1]. Clinical experience has demonstrated its remarkable therapeutic effects on various complicated diseases, including Covid-19; therefore, adoption of this system is spreading across clinics worldwide [2]. Among the thousands of traditional Chinese medicine (TCM) species, *Codonopsis pilosula* (Franch.) Nannf. (Campanulaceae) (*C. pilosula)* is one of the conventional herbs that is widely employed with considerable frequency and volume in more than 110 prescripts, and at least more than 1800 enterprises in China produce preparations containing *C. pilosula* [3]. Possessing notable therapeutic and modulating effects on lung-moistening, enhanced immune function, and anti-tumour activity, etc., the usage of dried roots of *C. pilosula* have been both extensively recorded in ancient Chinese medicinal documentaries and in authoritative modern medical regulations, such as the Chinese National Pharmacopoeia [4]. Apart from China, *C. pilosula* is also distributed in other Asian countries, especially in South Korea and Japan, however, China is the primary growing country with an increasing demand and planting area [4]. Owing to its functional components such as polysaccharides and saponins, *C. pilosula* has been listed as a food material with health and nutritional value by authorities in China since 2018. Nearly 200 healthy foods containing *C. pilosula* have been approved by the China Food and Drug Administration. As regulatory institutions increasingly acknowledge its medicinal and food-related characteristics, the demand and attention from scholars are expected to increase rapidly [4]. What's more, with gradual improvement of the international reputation of TCM and rapidly updated scientific research methods, as also that of *C. pilosula*, the study of TCM is accelerating rapidly in recent years [2]. In addition, to address the shortcomings of chemical synthetic drugs, natural medicinal plants are emerging quickly as having advantage of higher safety and multi-target point. At least 40 % of new drugs are estimated to developed from medicinal plants, however, the chemical constituents and pharmacological activities haven't been completely revealed for most of medicinal plants to date, and *C. pilosula* is no exception [5]. This means the research potential of *C. pilosula* in medicine and food field is huge, and with this trend, the related scientific literature is increasing annually.

The rapid development of scientific big data information analysis system has accelerated multidisciplinary research in all fields of TCM, such as the Traditional Chinese Medicine Systems Pharmacology Database and Analysis Platform (TCMSP) of China, with increasing number of chemical substance and pharmacodynamic values being revealed [6]. Similarly, scientific big data analysis of the published literature could provide important information in grasping popular research focus and trend, thereby speeding up the research and development for subsequent research [7]. Furthermore, analysis of past, current, and future trend is necessary to identify promising direction and dominant area of research field on *C. pilosula*. However, the majority of reported literature on *C. pilosula* are presented as research articles with a focus on chemical components or physiological effects, there were very few review articles conducted on *C. pilosula*. Anymore, conventional review articles are basically based on summary and analysis of existing literature with more subjective opinion of author, which usually lack of quantitative analysis on literature and interactive relations among information [8,9]. Hence, a new scientific and robust method should be introduced to analyze and predict the development of *C. pilosula*.

Bibliometric analysis has been exceptionally important for researchers to evaluate future developing trends and hot research directions in medicine or any field [6]. Such analysis can provide qualitative interpretation and analyze the evolution and dynamics of scientific information for a specific field by research productivity, progress, quality, impact and inter-connectivity of scientific works using mathematical and statistical methods [[9], [10]]. Therefore, bibliometric analysis could be an available way to grasp present situation and capture the developing trend of *C. pilosula* [8]. A large number of studies on *C. pilosula* have been conducted to date. However, no bibliometric study on this important herb has yet been reported. Hence, this study aimed to explore past and present situation and predict the future trend of *C. pilosula*-related research focus with comprehensive insights on the current status globally, which were based on data from Web of Science database in a quantitative way. Though numerous research studies have been conducted on *C. pilosula*, there is still a lack of bibliometric analysis on this significant herb. Based on data from the Web of Science database. This study will provide valuable insights for researchers, producers, and investors interested in *C. pilosula*, particularly those new to the topic, by highlighting key research institutions, countries, authors, emerging research areas, future directions in related fields.

## 2 Materials and methods

### 2.1 Source of the data and search strategy

All data were collected from the Web of Science (WOS) database [8]. In addition to WOS Core Collection (WOSCC) (1979-present), high-quality data source such as the Chinese Science Citation Database (CSCD) (1989-present) and Korean Journal Database (KCI) (1980-present) are also included in WOS database. The root of *C. pilosula* is widely used as important traditional medicines and OTC drugs in China, Korea, where it is distributed [4]. Therefore, the search scope in this paper considered the data source of CSCD, KCI, MEDLINE (1950-present), SciELO Citation Index (2002-present) and BIOSIS Citation Index (1994-present) included in the Web of Science database.

**Fig. 1:**
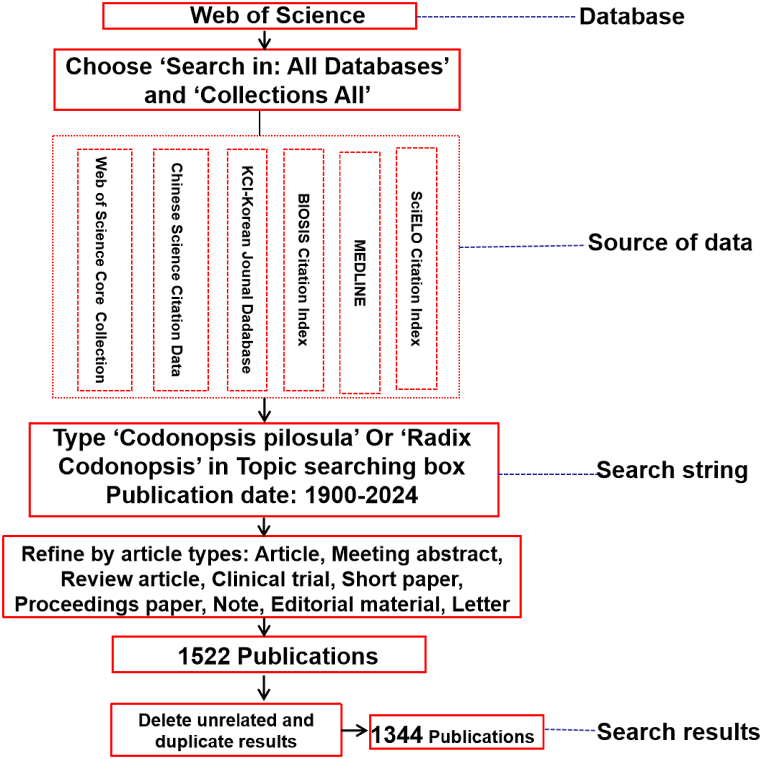
Search Strategy of the data in Web of Science.

The data search strategy adopted is illustrated in Fig. 1. The steps were as follows: go to find ‘Search in: All Databases’ and ‘Collections All’, then select Web of Science Core Collection, Chinese Science Citation Database, KCI - Korean Journal Database, MEDLINE, SciELO Citation Index and BIOSIS Citation Index. Time span: from 1900 to 01-01 to 2024-06-05. (data were collected on June 5, 2024). Type search string ‘Codonopsis pilosula’ in the Topic box, click ‘Add row’, choose ‘Or, Topic’ and type ‘Radix Codonopsis’ in the search box, and then click ‘Search’. A total of 1522 publications were identified. The results were categorised by publication type as article, review article, meeting abstract, proceeding paper, clinical trials, editorial material, note, short paper or letter; publications categorised as ‘others’ were removed as it was not clear as to which specific document type they belonged to. Finally, 1344 results were retained for further analysis. The literature was selected according to a careful reading of the keywords and abstracts and then downloaded into the Excel format (all literature information required was selected when saved), which was saved as the original information [11].

### 2.2 Data information collation and improvement

Owing to the inconsistency of the integrity of literature sourced from different data sources and different languages in the downloaded data, it is necessary to improve and acquire more relevant information in WOS database. The specific scheme of this step was: the 'UT (Unique ID)' column in the downloaded Excel data was found, use the screening function was used to classify and screen out information with Unique ID start with ‘WOS:, CSCD:, BCI:, etc.’, and copying the Unique ID number with same letters at the beginning. Then, the Unique ID numbers were pasted in the WOS database in the corresponding data source column to acquire the full record or more information as much as possible. To improve the information collected from WOSCC, the following steps were performed: find 'Search in: Web of Science Core Collection', find search by ‘Accession Number’ in the WOS database, then all copies of the Unique ID number beginning with ‘WOS:’ copied in Excel were pasted in Search bar, click and then selected all retrieved documents, select ‘export records’, and ‘Full record’ to Excel format. The literature information of other Unique ID numbers beginning with CSCD, BCI, KJD, MEDLINE and SciELO were collected according to the above method.

### 2.3 Removal of duplicate data

After the data was sorted out and completed, duplicate data were identified with COOC software and confirmed by reading the all information.

### 2.4 Conversion of synonym information

The unification of information with same meaning but expressed by using different keywords terminologies, author names, and organization names were performed by replacement function of COOC software. Using the frequency statistics function of the software, the duplication problem caused by the same meaning but individual letter case differences, synonyms, abbreviations, were checked one by one. And the repeated information was then collected into a replacement table, and the synonym merging function of COOC software was used to replace them with a single regulated word.

### 2.5 Conversion of information with different language

The information obtained in languages other than English in the screened list was translated into English using the CNKI translation tool before conducting further analysis.

### 2.6 Data format conversion

The compiled data form needs to be converted into text documents that can be read and analyzed by VOSviewer and Citespace using the data format conversion function of COOC software [11]. Text documents transformed by COOC were used by VOSviewer to draw a network visualization map with a Refworks format, by Citespace to draw Timeline View and Burstwords Map with a CNKI format [12,13].

### 2.7 Data analysis

The screened 1344 data was used for analyzing information including publication year, quantity, author, document type, affiliation, country, citation times, keywords, funding agencies and their interactive relationships by network analysis, keyword co-occurrence analysis, timeline view of keyword and burst word analysis. Data mining of network analysis and average appearing year were conducted by VOSviewer (version:1.6.18) [14]. The visualized figure by VOSviewer with links between nodes represent associations of each information, the strength of the link was expressed in terms of total link strength [11]. On visualized VOSviewer maps, different bubbles represent different elements, the size of each bubble indicates cooccurrence frequency, where the larger the frequency, the larger size of its bubbles; the larger the scale of the cooperation, the thicker of connecting line [15]. Citespace 6.1. R3 software was used to capture keywords associated with strong citation bursts and timeline view to predict frontiers and explore keywords’ co-evolutionary pathways [16].

## 3 Results and discussion

### 3.1 Publication overview

**Fig. 2:**
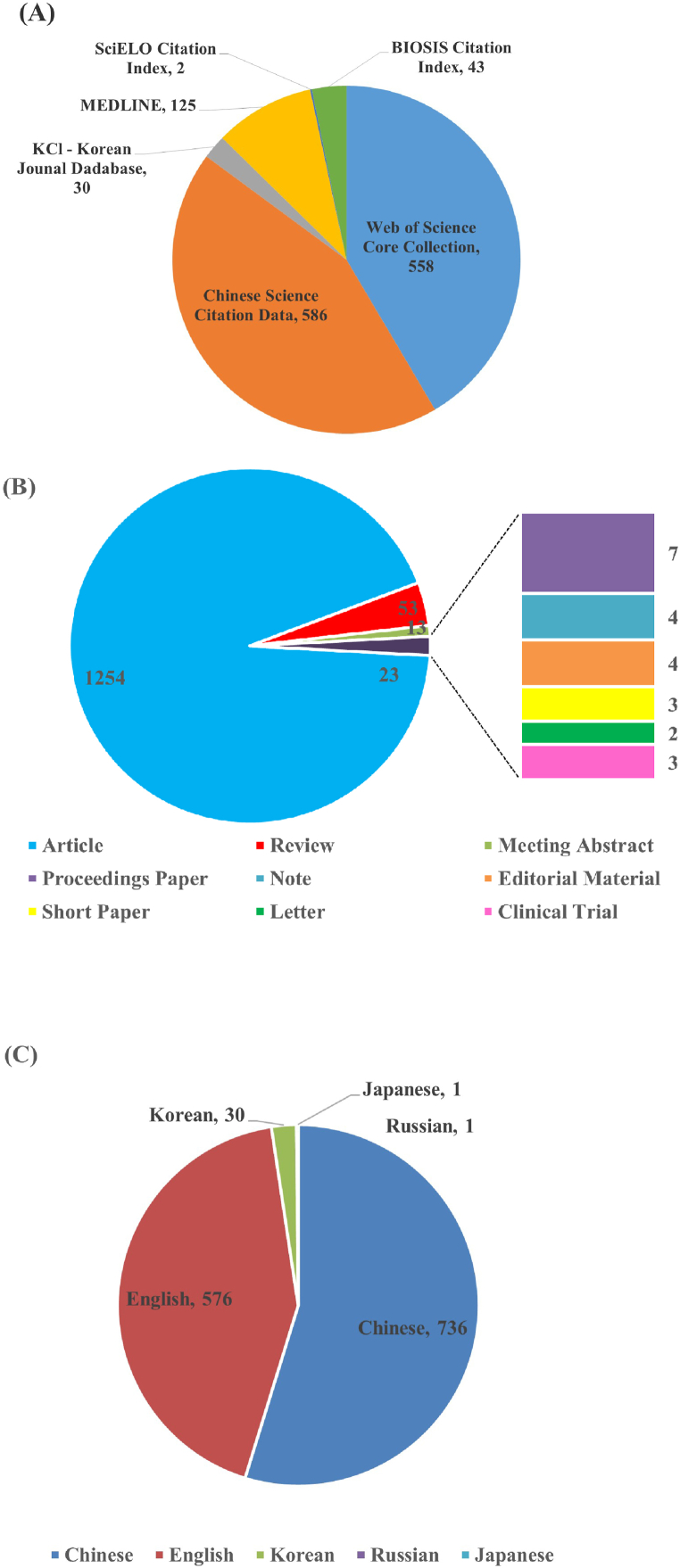
Overview information of refined 1055 publications. (A) Contribution of each information source of publications. (B) Document types of publications. (C) Writing language of publications.

Fig. 2 (A, B, C) provides an overview of the final list of 1344 publications retrieved, including publication output from each information source, document types and publication language. As shown in Fig. 2 (A), the highest contribution rate was obtained from the CSCD, at a high proportion of 43.60 %. Next, the Web of Science Core Collection contributed 558 (41.52 %) publications. Among the remaining databases, MEDLINE contributed 125 (9.30 %). BIOSIS Citation Index contributed 43 (3.20 %). KCI contributed 30 (2.23 %). SciELO contributed 2 (0.14 %). Fig. 2 (B) presents information on documents’ types, 1254 (93.30 %) of them are article papers, others include 53 review articles, 13 meeting abstract, 7 proceeding papers, 3 clinical trials, 4 editorial material, 3 short paper, and 2 letter. Fig. 2 (C) shows writing language of publications, 736 (54.76 %) were written in Chinese, only 576 (42.86 %) were written in English; 30 (2.23 %) were written in Korean, 1 was in Japanese and another 1 was in Russian.

Generally, WOSCC is considered as the world's leading citation databases with multidisciplinary information from over 18,000 high impact journals for at least 100 years, which has much higher reputation than CSCD database [15,17,18]. However, based on the above results, the CSCD database constructed by China since 1989 contributed much more articles on research of *C. pilosula* [15,17]. Thus, those publications written in Chinese (54.76 %) by Chinese scholars played a more dominating role in the global research field of *C. pilosula*. Obviously, the CSCD and WOSCC information source are the core publication source of WOS for the study of *C. pilosula*, but CSCD occupies an absolute dominant role. Therefore, a new finding in this study is that although the data collected by the CSCD in WOS is late, it still dominates, which reminds researchers of *C. pilosula* to realize the importance of CSCD information source in the future, these data included in it shouldn't be ignored. However, the CSCD library is mainly based on Chinese academic papers, the reading object is mainly Chinese researchers, which limits its influence in the world [17,18]. Additionally, from the language and information sources, it can be seen that Chinese scholars have an distinct advantage in global *C. pilosula* research, and the research form is mainly the publication of academic papers. This is related to the fact that the distribution of *C. pilosula* is mainly in China.

### 3.2 Publication output

**Fig. 3:**
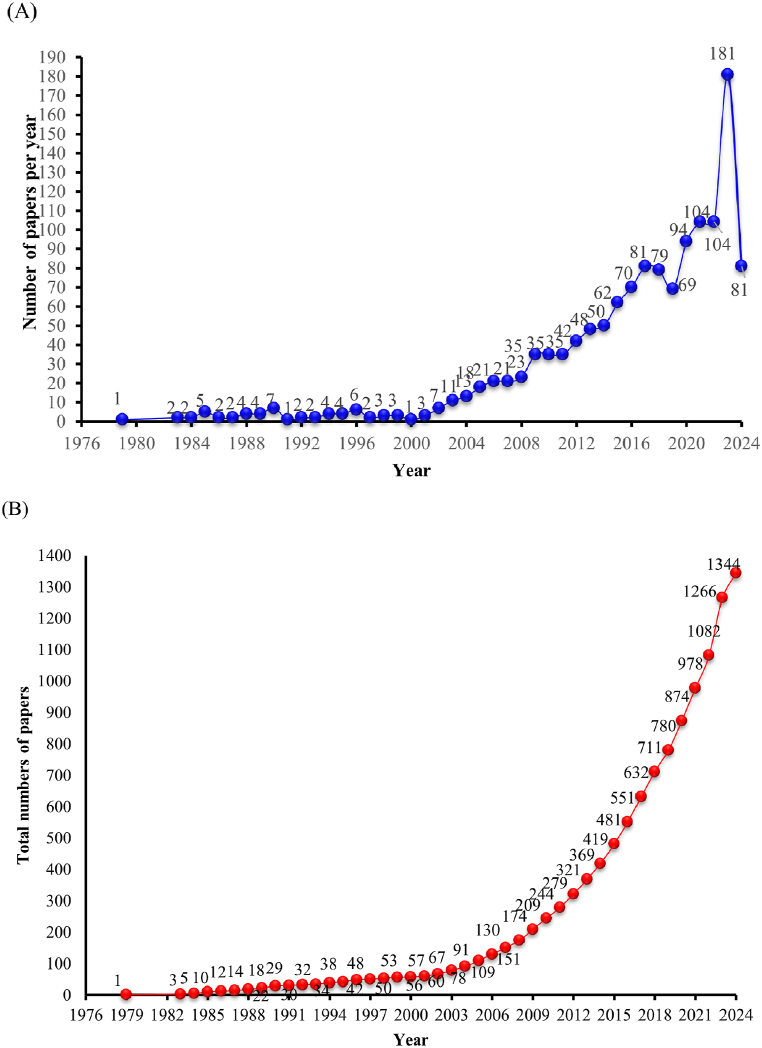
(A) Annual numbers of publications. (B) Cumulative numbers of publications.

The output of scientific literature is a mirror of research and development focus in a professional field. As it presents in Fig. 3(A and B), the research of *C. pilosula* is an expanding field presently, which have been continuing to attracting increasing attention of researchers over the past 20 years. The earliest research article on *C. pilosula* indexed in WOS was published in 1979, the annual output of publications has been rising straightly from 1979 to 2024 with a rapid increase until now. According to its developing trend of annual output and cumulative output, the research on *C. pilosula* has gone through 3 important stages: initial stage (1979–2002), fast development stage (2003–2012), and explosive development stage (2013-present). From Fig. 3 (A), at initial stage (1979–2002), there were only very few studies on *C. pilosula*. The average annual output number was 3.19. It is rather worth noting that the cumulative output of this stage from Fig. 3 (B) obtained a clear linear relationship with very good linear fitting characteristics (y = 3.2578x-6452.2, R^2^ = 0.9769). At fast development stage (2003–2012), the average annual number of publications reached 25.4. Large numbers of publications poured out with a relatively stable annual growth rate of 12.54–16.51 %, the average annual growth rate was 14.49 %. The cumulative output also got fine linear fitting characteristics (y = 26.897x-53817, R^2^ = 0.973) in this period. At explosive development stage (2013- present), the output increased sharply, reached as high as 85.25 per year (the output of 2024 was not calculated as the data of 2024 was get before June 5th). Until 2012, the cumulative output was only 321, but the publications emerged explosively after 2013, 69.57 % of output was contributed in this period. The average annual growth rate was 11.20 %. The cumulative output also obtained considerable linear fitting characteristics with R^2^ of 0.9935, (y = 81.908x-164528, R^2^ = 0.9721). From the above, it can be seen that each developing period of *C. pilosula*'s research experienced a fast speed by linear relationship with very good linear fitting characteristics naturally, of which the correlation coefficient of R^2^ reached as high as 0.9721–0.9769. Usually, when R^2^ > 0.99, we consider the fitted line meet requirement with ideal linear fitting characteristics. The closer the R^2^ value approaches to 1, the higher the degree of agreement to a fitted linear line [20]. Thus, there's enough proof to certify that the speed of development of *C. pilosula*'s research field is staggering.

The reason for explaining low output before 2000 may attribute to possible reasons of low and inadequate availability or change of the scope included for publications' information in the database, especially in early years [12,[19], [22]]. Another reason for thriving development after 2000 could may have strong relationship with the promulgation of Chinese Materia Medica Development (2002–2010), Chinese medicine grasped first opportunity for policy and funding support, the related scientific research attracted more attention than before in 2002–2010 [21]. In 2015, with implementation of Chinese Medicine Law and Nobel Prize awarded to Chinese medical researcher Youyou Tu, the development of modern Chinese medicine got a new milestone [23]. As a result, under this background, the scientific research of *C. pilosula* developed much faster with a sharper increase with the promotion and development of Chinese medicine in the whole world. Until now, as trend of publication's output from Fig. 3 suggests, the development of *C. pilosula*'s research is still at a stage with rapid increase. Therefore, this plant should be a hot research material both at present and in the near future with quite promising prospect. More over, as a favorable medicinal plant for medicine, the demand and further research could increase continually. In the new era, the research of natural plant components is an important direction for the development of new drugs [21]. Based on its long medicinal history, rich clinical verification and literature data, the plant is expected to make new breakthroughs in the future research and development of new drugs.

### 3.3 Countries and institutions

**Fig. 4:**
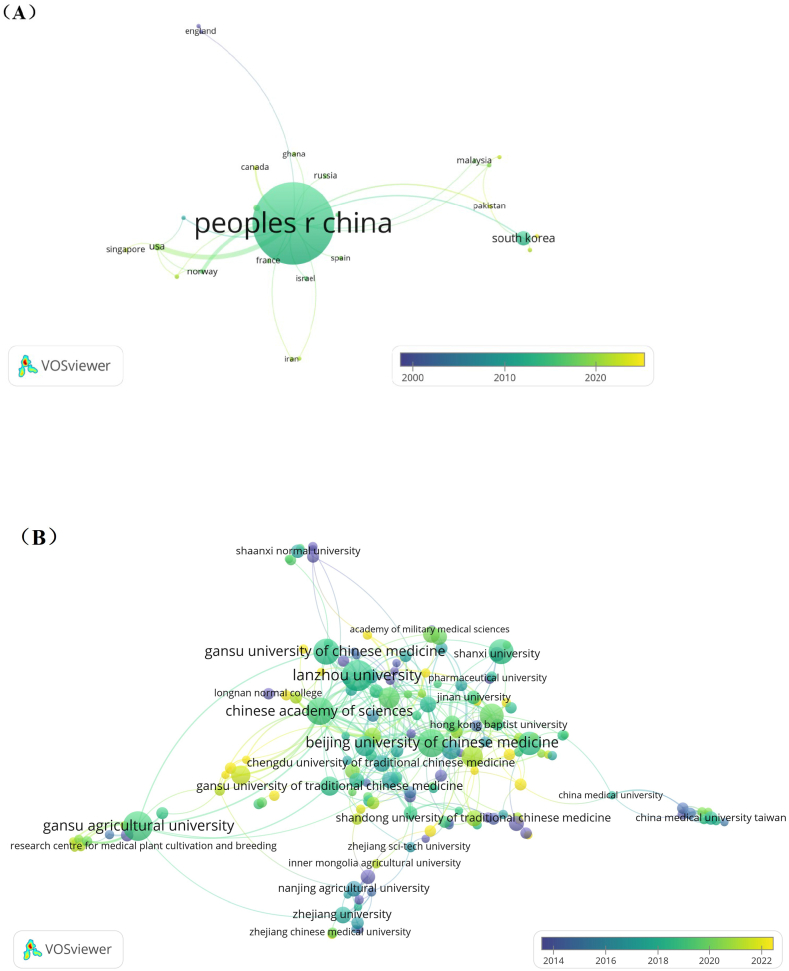
(A) Overlay map of network and time of collaboration among countries. (B) Overlay map of network and time of collaboration among institutions.

The publications were contributed by totally 1292 institutions from 27 countries. The visual analysis of country and institution were conducted by VOSviewer in Fig. 4(A and B). The countries occurred over 1 time and institutions occurred over 3 times were screened for co-occurrence analysis. Finally, 27 countries and 187 institutions meet threshold and formed network map. The statistical results analyzed by VOSviewer of Top 10 countries and institutions are listed in Table 1.

**Table 1:** Top 10 contributed countries, institutions, total link strength with each other and their average publication years.

	Rank	Name of country/institution	Output (% of 1344)	Total link strength	Average publication year
Country	1	Peoples R China	992 (73.81)	48	2014
	2	South Korea	49 (3.65)	5	2013
	3	USA	14 (1.04)	16	2019
	4	Japan	12 (0.89)	7	2017
	5	Norway	8 (0.60)	10	2017
	6	Australia	5 (0.37)	3	2017
	7	Malaysia	4 (0.30)	3	2009
	8	Russia	4 (0.30)	1	2021
	9	Singapore	4 (0.30)	5	2019
	10	Canada	3 (0.22)	3	2014
Institution	1	Lanzhou University	67 (4.99)	27	2018
	2	Gansu Agricultural University	58 (4.32)	34	2019
	3	Beijing University of Chinese Medicine	52 (3.87)	55	2019
	4	Chinese Academy of Sciences	49 (3.65)	21	2019
	5	Gansu University of Chinese Medicine	44 (3.27)	33	2019
	6	Shanxi Medical University	39 (2.90)	9	2019
	7	Nanjing University of Chinese Medicine	35 (2.60)	27	2020
	8	Guangzhou University of Chinese Medicine	33 (2.46)	5	2019
	9	Shanghai University of Traditional Chinese Medicine	27 (2.01)	33	2021
	10	China Academy of Chinese Medical Sciences	25 (1.86)	6	2019

Note: Output (% of 1344) represents the numbers of publications, the numbers in the bracket represent the percentage in all 1344 publications. Total link strength and average publication year were calculated by VOSviewer involved in saved CSV file.

The map showing countries' connection by line can express the degree of cooperation among countries in a certain field, which provides a concentrated information for assessing or mining scientific information for researchers [23]. For scientific research of *C. pilosula*, China occupies dominant position for contributing 992 (73.81 %) publications independently and cooperated with other countries for an average publication year of 2014. Then is South Korea, contributed 49 (3.65 %); United States contributed 14, Japan contributed 12, Norway contributed 8, Australia contributed 5; others contributed 3–4. Fig. 4 (A) is the overlay map visualization of network and time of collaboration among countries. Evidently, China stands at a core position on cooperating with Asia, Africa and Europe countries, the total link strength with other countries reaches 48. China cooperated more studies with United States (link strength:16), Norway (link strength: 10) and Japan (link strength: 6) than others. The highest cooperation intensity forms between China and United States (link strength:16). What's more, judging from map and Table 1, United States, Singapore, Russia and Canada joined into this research field more recently with average publication years after 2017. Although South Korea has more research and started work earlier than others except China with an average publication year of 2013, its cooperation with foreign countries is not strong (link strength: 5).

The analysis of institutional cooperation can provide information on organizations with high contributions in the analyzed field [24]. Fig. 4 (B) is overlay map visualization of network and time of collaboration among institutions. Table 2 is the statistics of Fig. 4 (B), which lists Top 10 contributing countries and institutions with their total link strength of each other. Lanzhou University contributed most publications with a number of 67 (4.99 %). Then is Beijing University of Chinese Medicine of 58 (4.32 %), and Chinese Academy of Sciences of 52 (3.87 %). Of top 5 institutions, 3 of them including Lanzhou University, Gansu University of Chinese Medicine and Gansu Agricultural University are all affiliated to Gansu Province, where is currently the largest *C. pilosula* producing area in China. The average publication year from Table 1 and Fig. 4 (B) by Chinese University of Hong Kong (2011) is much earlier than others. However, the average publication years of other Top 10 institutions were all around 2019–2020. This provides an information that the year 2019–2020 is an important period for researchers of *C. pilosula* emerged recently. Moreover, the complex connecting lines in visualization network map reveals a good cooperation relationship among institutions in this field, among them Chinese Academy of Sciences established the highest cooperation intensity of 55 with other institutions.

**Table 2:** Top 10 productive authors in research of *Codonopsis pilosula* (Franch.) Nannf. (Campanulaceae).

Rank	Author's name	Author's institution	Output (% of 1344)	Total link strength	Average publication year
1	Fangdi Hu	Lanzhou University	48 (3.05)	202	2020
2	Jianping Gao	Shanxi Medical University	28 (2.08)	97	2018
3	Yuan Chen	Gansu Agricultural University	21 (1.56)	64	2019
4	Fude Yang	Gansu University of Chinese Medicine	18 (1.34)	52	2021
5	Jiankuan Li	Shanxi Medical University	16 (1.19)	62	2019
6	Yuanfeng Zou	Sichuan Agricultural University	15 (1.12)	128	2016
7	Fengxia Guo	Gansu Agricultural University	13 (0.97)	45	2018
8	Ruibin Bai	Lanzhou University	12 (0.89)	64	2019
9	Yan Wang	Lanzhou University	12 (0.89)	45	2020
10	Faming Wu	Zunyi Medical University	11 (0.82)	39	2018

Note: Output (% of 1344) represents the numbers of publications, the numbers in the bracket represent the percentage in all 1344 publications. Total link strength and average publication year were calculated by VOSviewer involved in saved CSV file.

These results show that the research of *C. pilosula* has been carried out in 27 countries worldwide, and has attracted global attention and established in a cooperative network relationship with China occupies a core position. As the second contributing country, South Korea has also done considerable work. Because Gansu Province in China is the main producing area of *C. pilosula*'s artificial cultivation, several institutions in Gansu Province involved in agricultural cultivation, traditional Chinese medicine and comprehensive research have done substantial work. Therefore, Gansu province in China belongs to the concentrated area of *C. pilosula*'s research and have made important contributions to the development and research of *C. pilosula*, and has an absolute advantage in the construction and research of the production area. Many institutions, from Gansu and Shaanxi in the northwest of China to Zhejiang and Nanjing in the southeast coast, in addition to universities, as well as famous research institutions such as the Chinese Academy of Sciences have obtained many fundamental achievement in this field. As it also distributes in other Asian countries including South Korea and Japan, South Korea occupies 2nd dominating role in *C. pilosula'*s research, but Japan did only few work in this herbal medicine. Thus, *C. pilosula* is a popular medicine arisen high attention with considerable research value worldwide involved in thousands of authoritative institutions.

### 3.4 Authors

**Fig. 5:**
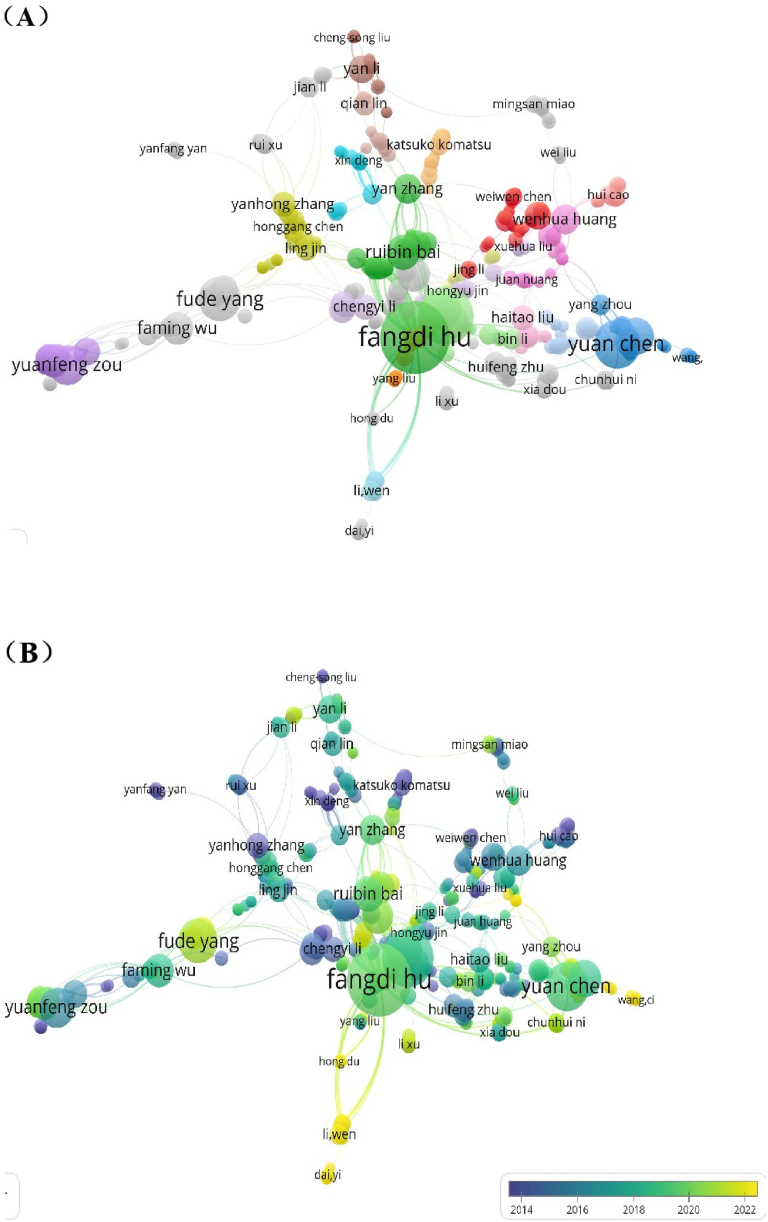
(A) Network map of authors by cluster. (B) Overlay map of network and time of collaboration among authors.

The publications were contributed by 6234 authors. The analysis of author co-occurrence was performed with VOSviewer. Authors with a minimum of three publications were screened for analysis. Finally, 371 authors met the selection criteria and a network map of the results was constructed. Fig. 5 (A) is the network visualization of authors by cluster (showing in different colors). The authors were clustered into 18 groups, suggesting the presence of 18 collaborating research groups on *C. pilosula*. The productivity of an author in a research area is one of the significant indicators to determine his influence in that field [23]. Among all the main research groups, Fangdi Hu from Lanzhou University gained highest frequency, and the statistics are listed in Table 2. She is not only the most productive author (48) but also had the strongest link strength (202) with others, who has a significant influence in this field. Then is Jianping Gao (28) from Shanxi Medical University. Yuan Chen, Fude Yang and Jiankuan Li (21, 18, 16) also contributed relatively more publications than others. Fig. 5 (B) is the overlay visualization of network and time of collaboration among authors. Based on Fig. 5 (B) and Table 2, the average publication years of Top 10 authors are in a range of 2016–2020. Interestingly, all of these authors are from China. Nevertheless, from Table 1, the average publication year of China is 2014, thereby, the Top 10 authors from China are all involved in the study of *C. pilosula* quite recently, they all play important roles in this field and are also be the leading authors of later research in the near future. Several researchers emerged in this field around 2022, one of impressive researcher is FudeYang, who is mainly engaged in Chinese medicine. This has a close relationship with the rapid development and attention of traditional Chinese medicine in recent years and the emergence of new pharmacological methods of traditional Chinese medicine. Because *C. pilosula* is one of the important tonic medicines in traditional Chinese medicine with large dosage in use, and the cultivation has realized large scale of artificial technology. At the same time, from Fig. 5, the research of *C. pilosula* were completed by several fixed large teams including Fangdi Hu, Yuan Chen, Fude Yang, and Yuanfeng Zou. Fangdi Hu has made a systematic study on the pharmacology, cultivation, resource development and utilization, processing and storage of *C. pilosula*, who has made a prime contribution to the research and production by combining Fude Yang of Gansu University of Traditional Chinese Medicine and Yuan Chen of Gansu Agricultural University. Though Stable cooperation relationship has been established among most of these team, the overall cooperation network of all teams is not strong, because most of the teams only have stable cooperation with fixed teams, leading to the rest of other teams have little cooperation. But Hu Fangdi and Chen Yuan's team have more cooperation with the rest of teams with larger contributions. This may because the specific research directions of the researchers are quite different. As far as the research direction and field of researchers are concerned, the research fields of *C. pilosula* include: agricultural production, medicine, and other comprehensive research. Therefore, the cooperation among researchers has resulted in obvious gap of the network of different teams. According to the analysis results of author, the top 3 experts in the research field of *C. pilosula* are: Fangdi Hu, Jianping Gao and Yuan Chen, who are specialists in Chemical analysis and Pharmacological research of Traditional Chinese medicine, Standard and Quality research of Traditional Chinese medicine, Cultivation and Production research, respectively. These three experts are authoritative and are the leaders and have important position in *C. pilosula*'s field.

### 3.5 Highly cited publications

The most important indicator in a research field is ‘most cited documents’, which is usually measured by a count of the number of citations. Highly cited papers are regarded as key papers which can be used to analyze the theoretical basis of a topic and study methodology in a field with high academic influence [24]. The Top 10 publications with the most highly cited citations in research on *C. pilosula* are listed in Table 3. All of them were written in English, 7 are research articles and 3 are review papers. The cited times of the highest cited publication is 158, it was conducted by Qiqihar Medical University of China published in International Journal of Biological Macromolecules in 2008, which mainly focused on polysaccharide's structure and immunity activity of *C. pilosula*. Only 3 publication gained high cited times published in recent 10 years. Moreover, of Top 10 highly cited publications, Journal of Ethnopharmacology contributed most documents (4, 40 %), and 7 of them studied on the pharmacological activity of *C. pilosula*. Among all the 10 publications, 4 of them studied polysaccharide from *C. pilosula.* Polysaccharide is a core bio-active medicinal components, and has arisen wide concern on pharmacological researchers. The above published literature have played leading roles in the study of polysaccharides and pharmacology effects of *C. pilosula*, and provides an important reference for subsequent articles in terms of research methods and ideas.

**Table 3:** Top 10 most cited publications in research of *Codonopsis pilosula* (Franch.) Nannf. (Campanulaceae).

Published year	Title	Total cited times	Published Journal	Affiliations	Countries	Language	Document type
2008	Structural characterization of a water-soluble polysaccharide from the roots of Codonopsis pilosula and its immunity activity	158	International journal of biological macromolecules	Qiqihar Medical University	China	English	Article
2018	Traditional uses, phytochemistry, pharmacology and toxicology of Codonopsis: A review	135	Journal of ethnopharmacology	Chinese Academy of Medical Sciences, Peking Union Medical College	China	English	Review
2011	Evaluation of antidiabetic potential of selected traditional Chinese medicines in STZ-induced diabetic mice	134	Journal of ethnopharmacology	Southwest University	China	English	Article
2012	Immuno-enhancement effects of Shenqi Fuzheng Injection on cyclophosphamide-induced immunosuppression in Balb/c mice	133	Journal of ethnopharmacology	Sun Yat Sen University	China	English	Article
2015	The genus Codonopsis (Campanulaceae): a review of phytochemistry, bioactivity and quality control	131	Journal of natural medicines	Chinese Academy of Sciences; University of Toyama	China, Japan	English	Review
1999	Stimulating activity of Chinese medicinal herbs on human lymphocytes in vitro	119	International journal of immunopharmacology	University of Occupational & Environmental Health	Japan	English	Article
2021	Extraction, purification, structural characteristics and biological properties of the polysaccharides from Codonopsis pilosula: A review	113	Carbohydrate polymers	Chengdu University of Traditional Chinese Medicine; Zunyi Medical University	China	English	Review
2010	Application of response surface methodology for optimization of polysaccharides production parameters from the roots of Codonopsis pilosula by a central composite design	113	Carbohydrate polymers	Qiqihar Medical University; University of Birmingham	China, England	English	Article
2004	The antioxidant effects of aqueous and organic extracts of Panax quinquefolium, Panax notoginseng, Codonopsis pilosula, Pseudostellaria heterophylla and Glehnia littoralis	112	Journal of ethnopharmacology	Chinese University of Hong Kong; Nankai University; China Agricultural University	China	English	Article
2013	Structural characterization and antitumor activity of a pectic polysaccharide from Codonopsis pilosula	110	Carbohydrate polymers	Lanzhou University	China	English	Article

Note: the cited times in above table are total cited times of all information source recorded by WOS database.

### 3.6 Analysis on keywords and research focus

#### 3.6.1 Knowledge map of keywords

Keywords are words extracted from text that can reflect the main idea of article [24]. The analysis of the co-occurrence knowledge map and evolution of high-frequency keywords can visually reflect the evolution trend of research frontiers and hotspots in a field [25]. To discover past and present research focus and identify research frontier of *C. pilosula*'s research, the co-occurrence network map of keywords, timeline view and burstiness of keywords over time were analyzed by VOSviewer and Citespace software.

**Fig. 6:**
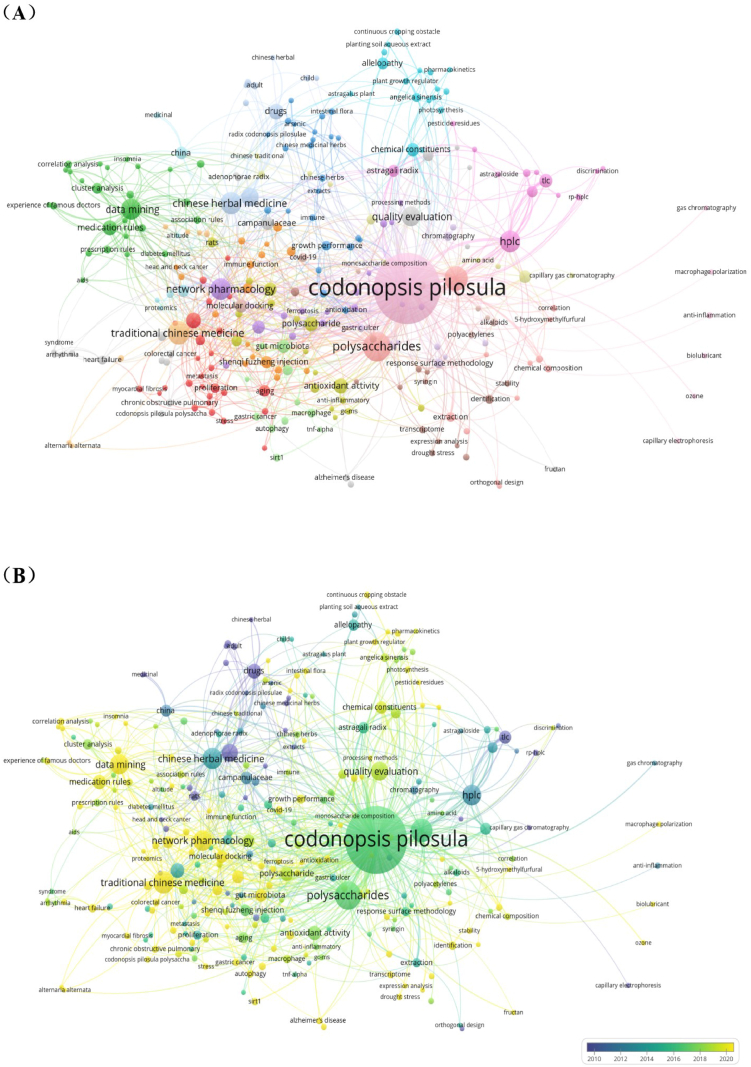

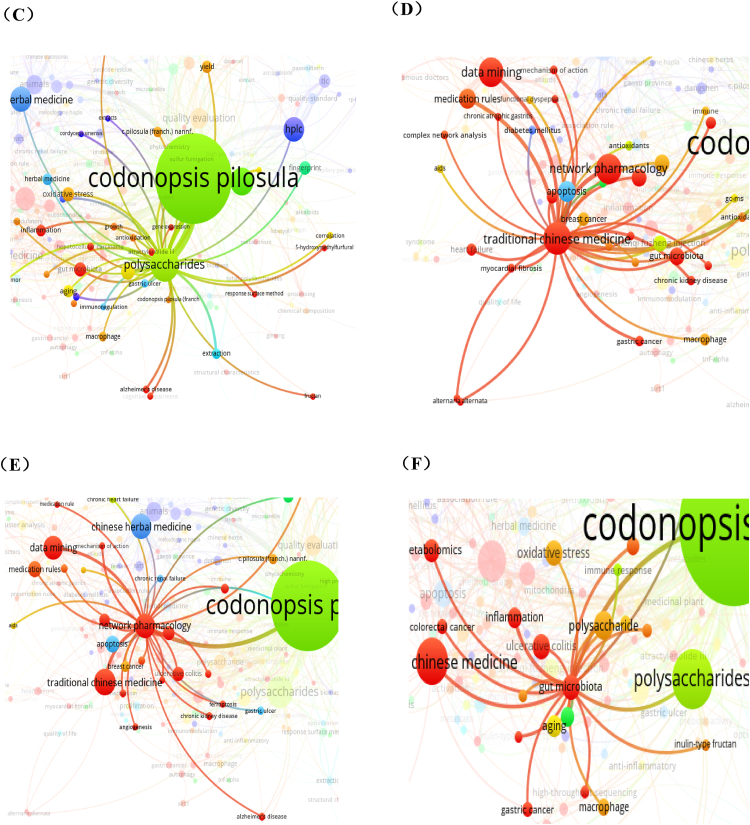
Analysis of keywords' co-occurrence related to *Codonopsis pilosula* (Franch.) Nannf. (Campanulaceae) research. (A) Cluster network map of keywords. (B) Overlay map of network of keywords and time. (C) Analysis of keywords' co-occurrence of ‘polysaccharides’. (D) Analysis of keywords' co-occurrence of ‘traditional Chinese medicine’. (E) Analysis of keywords' co-occurrence of ‘network pharmacology’. (F) Analysis of keywords' co-occurrence of ‘gut microbiota’.

Fig. 6 shows a visualization of the co-occurrence network map as clusters and an overlay of the co-occurrence network map of keywords and time by VOSviewer. Totally, 3883 keywords were identified. The keywords occurred at least 3 times were screened for analysis through full counting method, 292 keywords meet threshold and formed network map. Fig. 6 (A) is cluster visualization of keywords' co-occurrence, the different color indicates different clusters, and the cluster construction is based on relationship among the items resulting in groups of closely related elements of co-occurrence calculated by software. The keyword of *Codonopsis pilosula* occurred most with 437 times. The keywords expressed with big nodes in Fig. 6 (A) mainly includes polysaccharide, lobetyolin, data mining, Chinese herbal medicine, HPLC, network pharmacology, medication rules, apoptosis, animals, astragali radix, etc. These keywords are mostly related to studies on chemical constituents, quality control, pharmacological activity and data mining. Fig. 6 (B) is overlay map network of keywords' co-occurrence and time. In this map, different color represents different time for the corresponding keywords. In different developing period, the researchers' focus varied over time. In earlier time, the purple nodes in the map suggests (before 2010) the research focus was mainly on pharmacological activity used as Chinese herbal medicine, and animal studies were a hot method at that time. Later on, the keywords were mainly related to chemical analysis on quality standard, components and extracts, mainly including TLC, HPLC, fingerprint, polysaccharide, lobetyolin, etc. After 2018, the nodes in light green and yellow suggests that new scientific research approach and areas were introduced to this field, including gut microbiota, alzheimer's disease, metabolormics, selenizing polysaccharide, UPLC-MS/MS, factor analysis, mediation rules and data mining, etc. From the above, the research focus of *C. pilosula* varies in different time, with evaluation and emergence of new scientific techniques, the research focus also evolute over time. Among all the keywords with notable frequency in Fig. 6, polysaccharide gained highest frequency of 75, it is a hot focus as a main active ingredient for medical use. As shown in Fig. 6 (C), the research of polysaccharides mainly focus on inflammation, oxidative stress, Alzheimer 's disease, aging, extraction, gut microbiota and other research topics. Then is the keyword of ‘lobetyolin’, it is a unique active ingredient of *C. pilosula*, which is a key component for quality control of *C. pilosula*, and the research frequency reached as high as 53. Keyword of ‘traditional Chinese medicine’ gained 3rd frequency in this field, as shown in Fig. 6 (D), the effects on cancer, AIDS, heart failure, chronic kidney disease, and other disease were correlated to this herb. Moreover, data mining and network pharmacology are regarded as hot focus in traditional Chinese medicine research of *C. pilosula.* As shown in Fig. 6 (E), network pharmacology is also a notable focus, especially for medication rules and research on disease. As shown in Fig. 6 (F), gut microbiota, a hot research focus in science worldwide has also become a focus of *C. pilosula*, the related keywords are mainly connected to polysaccharides and pharmacological effects.

#### 3.6.2 Timeline view of the keywords

**Fig. 7:**
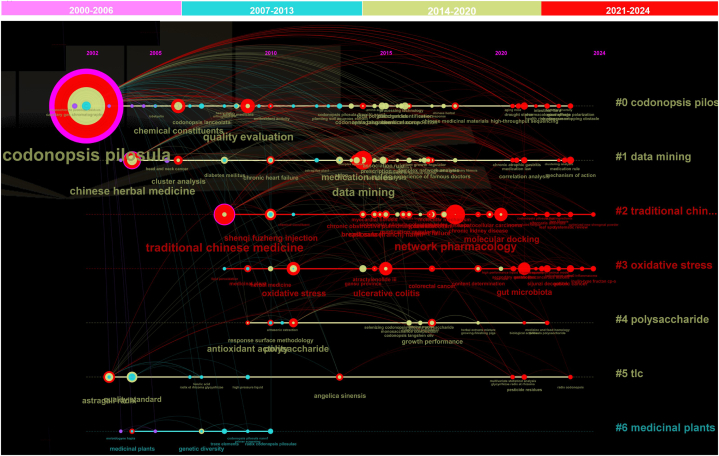
Timeline view of keywords clustering related to *Codonopsis pilosula* (Franch.) Nannf. (Campanulaceae) research.

Fig. 7 shows a timeline view of keyword clustering related to *C. pilosula*'s research since 2000 by Citespace, and demonstrates the evolutionary change in keywords over time. The Top 6 clustered keywords with frequency over 3 from 2000 to 2014 are shown in this picture. The size of the nodes reflect the frequency of keywords. The color of the nodes changed from 2000 to 2024 with color of rainbow, when the color of the nodes approximates to red (2021–2024), yellow green (2024–2020), sapphirine (2007–2013) and mauve (2000–2006), the appearing year of the keyword corresponds to the color. The connection line of keywords means that they have mutual connection, the color of the line represents time, and the thickness of the line represents the degree of association.

According to the colors, nodes, and connection lines in the picture, the notable nodes with high frequency of cluster 0 in yellow green color of central part and red color in rest part representing research focus on ‘chemical constituents’ and ‘quality evaluation’, and have connecting line with other smaller nodes in this cluster, which are mainly for pharmacological effects and processing. This suggests the chemical components and quality control are 2 important factors for present research from 2014 to now.

Cluster 1 of ‘data mining’ has notable nodes of ‘Chinese herbal medicine’, it started from 2000 and gained increasing frequency over time till present. Then is ‘medication rule’, started in 2014, with connecting line of ‘medicine medication law’, ‘correlation and clustering analysis’. Then is ‘data mining’, developed around 2014 with research focus on mechanism, correlation and clustering analysis. Among all nodes in this cluster, ‘experience of famous doctor’ and ‘complex network analysis’ raised high attention since 2014–2020.

Cluster 2 of ‘traditional Chinese medicine’ has most numbers of red nodes, suggesting research on this direction is now a hot research focus with very high attention. Since 2007, traditional Chinese medicine has been gaining increasing attention with frequency of 4–31 publications/year, more than 11 new research directions have been derived from this field with considerable attention. The research on ‘shenqifuzheng injection’, ‘breast cancer’, ‘lipid bimetallism’ and other keywords belongs to disease gained notable frequency since 2014 to present. This means the medicinal function for treating various disease is now still an important research direction, more over, the output of the study and the importance of this plant could continue to increase. Keywords of ‘network pharmacology’ and ‘molecular docking’ acquiring frequency of 35 and 12 stared from 2014 to present. This 2 keywords gained close connection lines with former and later research of this cluster, showing that network pharmacology and molecular docking have become an important research factor widely considered in research on traditional Chinese medicine.

Cluster 3 with big nodes of ‘ulcerative colitis’ and ‘gut microbiota’ showed close connecting line with ‘oxidative stress’. What's more, they are connected to ‘traditional Chinese medicine’ in cluster 2 and ‘polysaccharides’ in cluster 4. This may attribute to the notable function of *C. pilosula* on treating enteric disease by modulating oxidative stress with polysaccharides as a main component. ‘Oxidative stress’ emerged in 2007 in this field, and developed fast with presence of ‘gut microbiota’, and also connected closely to ‘intestinal cancer’ and related disease. This means *C. pilosula* is also used for treating intestinal cancer by modulating oxidative stress. Keyword of ‘gut microbiota’ started in 2021 and gained high frequency of 21, suggesting it has become a new and very hot research focus. Then, several new direction with small scale emerged since 2021 in this cluster.

Cluster 4 shows keywords of main active components of *C. pilosula,* including ‘growth performance’ and extracting method of ‘response surface methodology’. The nodes mainly started in 2007, among them, ‘antioxidant activity’ as the bio active effects is the most notable node with attention to present, but has more frequency in 2014–2020. And ‘medicine and food homology’ connecting to ‘polysaccharides’ has been a new focus since 2021. This has important relation for *C. pilosula* has been listed as food material with healthy and nutritional value by authorities of China since 2018.

In cluster 5, ‘astragali radix’ acquires frequency of 1–5 since 2000. According to the connecting line with other keywords, this keyword is closely related to ‘network pharmacology, ‘molecular docking’ used in traditional Chinese medicine, this is due to the frequent combination with *C. pilosula* and another keyword of ‘angelicae sinensis’ for medication of traditional Chinese medicine. The combination with these 2 medicine for treating disease is also a research focus presently. Then is ‘quality standard’ gained high attention from 2000 to 2014, but gained few attention after 2014, which can also been read from the color of the node. This implies that ‘quality standard’ was once an important research direction, but when the related work was completed, it is not a focus no longer. This indirectly shows that the quality standard of *C. pilosula* has established maturely.

Cluster 6 named of ‘medicinal plants’ shows notable keywords of ‘medicinal plants’ and ‘genetic diversity’, this 2 keywords belong to the prime cognition knowledge of this plant, which were mainly studied around 2000, and the small nodes in sapphirine color imply that the cognition of *C. pilosula* has been known to scientists formely, but not focus any more.

From the whole timeline view, the research of *C. pilosula* has undergone changes in multiple research directions from 2000 to 2024. From the improvement of quality standards around 2000, the genetic diversity and basic cognition of species, to the study of active ingredients mainly composed of polysaccharides, the combination usage of *Astragalus* and *Angelica*, and the active ingredients, to the study of pharmacological effects and mechanisms of regulating oxidative stress in recent years (after 2020), the regulation of gut microbiota, and the big data mining of traditional Chinese medicine based on network pharmacology and medication rules, all these scientific work have been completed have derived many new research directions. In a word, cluster 2 and 3 are both hottest research directions with many new focus, and the research on the pharmacodynamic effects, mechanism and data mining of traditional Chinese medicine have derived several new research hotspots, which should be the mainstream direction of future research. In addition, the research on effects and mechanisms related to oxidative stress, especially the regulation of cancer and intestinal diseases, has become another mainstream direction. Moreover, quality evaluation and chemical composition are still important factors in study of *C. pilosula*. However, the improvement of quality standards and the cognition as medicinal plant are no longer research focus of the plant. It is worth noting that frontier research, such as the study of gut microbiota, has been integrated into it, which proves that *C. pilosula* has received enough attention in the frontier research, keeping pace with the times.

#### 3.6.3 Burst words of keywords

**Fig. 8:**
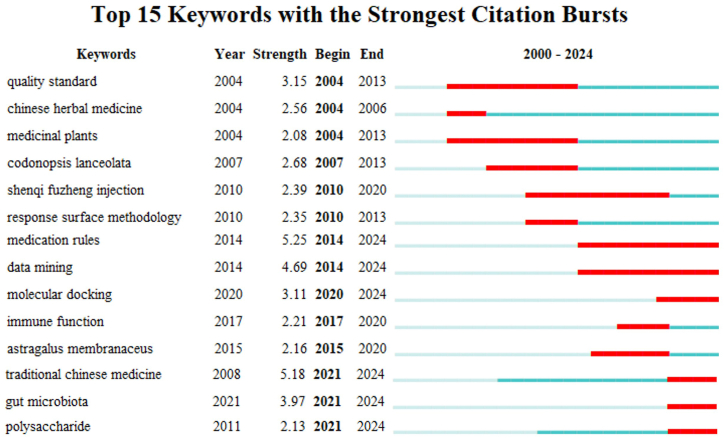
Top 15 keywords with the strongest citation bursts related to *Codonopsis pilosula* (Franch.) Nannf. (Campanulaceae) research.

Burst words refer to the keywords experienced a significant increase in frequency and intensity in a certain period of time. Keyword burst analysis can intuitively characterize the transfer and evolution of research focus, hotspots and frontiers in this field [11]. Fig. 8 is the Top 15 keywords with the strongest citation bursts calculated by Citespace software since 2000–2024. In this picture, the red bar in green line represents lasting period of corresponding listed words occurred with high frequency. Corresponding to previous analysis, the research in this field mainly focused on establishing of quality standard and the usage in Chinese herbal medicine in early stage from 2004 to 2013, and the basic cognition knowledge as medicinal plant. Because *C. pilosula* is often used in combination with *Astragalus* in traditional Chinese medicine, the word of *Astragalus membranaceus* also appears with association of *C. pilosula* for a long time in 2015–2020. With the development of evidence-based medicine and the emergence of big data analysis like TCMSP, the research of association rule, traditional Chinese medicine, data mining, mediation rule, network pharmacology and molecular docking have become hot points in *C. pilosula*'s research from 2014 to 2024. Apparently, these research hotspots are frontiers presently, including. All of these 6 keywords are proposed to be lasting in the near future, and could soon become main subjects of this field in short time. The research related to the pharmacodynamic effects, mechanism and data mining of traditional Chinese medicine and oxidative stress should be mainstream direction in present and near future.

The above analysis of keywords were analyzed by knowledge map from VOSviewer, timeline view and burst words from Citespace. These visualization methods express consistent information in terms of core research directions and frontier focus. However, each method has its own advantages in data display [11,13], knowledge map shows the network relationship among keywords, timeline view shows the derivation path and trend of research hotspots and research directions over time, burst word shows the change law of research frontiers in each developing period and can detect current frontier hotspots. The results of 3 visualization methods all suggest the keywords of *C. pilosula*'s research are mostly related to studies on pharmacological activity, chemical constituents, quality control, antioxidant activity, biological activities and main active component of polysaccharide and data mining research. The research focus of *C. pilosula* varies in different time. Timeline view and burst words detection suggests the research of medication rules, data mining, molecular docking, traditional Chinese medicine, gut microbiota, and polysaccharide have become hot focus and frontier focus presently and in the near future. The new focus of worldwide frontier research area of molecular docking and gut microbiota have also become new focus in research on *C. pilosula*, and should be also frontiers in the near future. This implies that with the evaluation and emergence of new scientific techniques, the research focus also evolute over time quickly.

### 3.7 Funding agencies

**Fig. 9:**
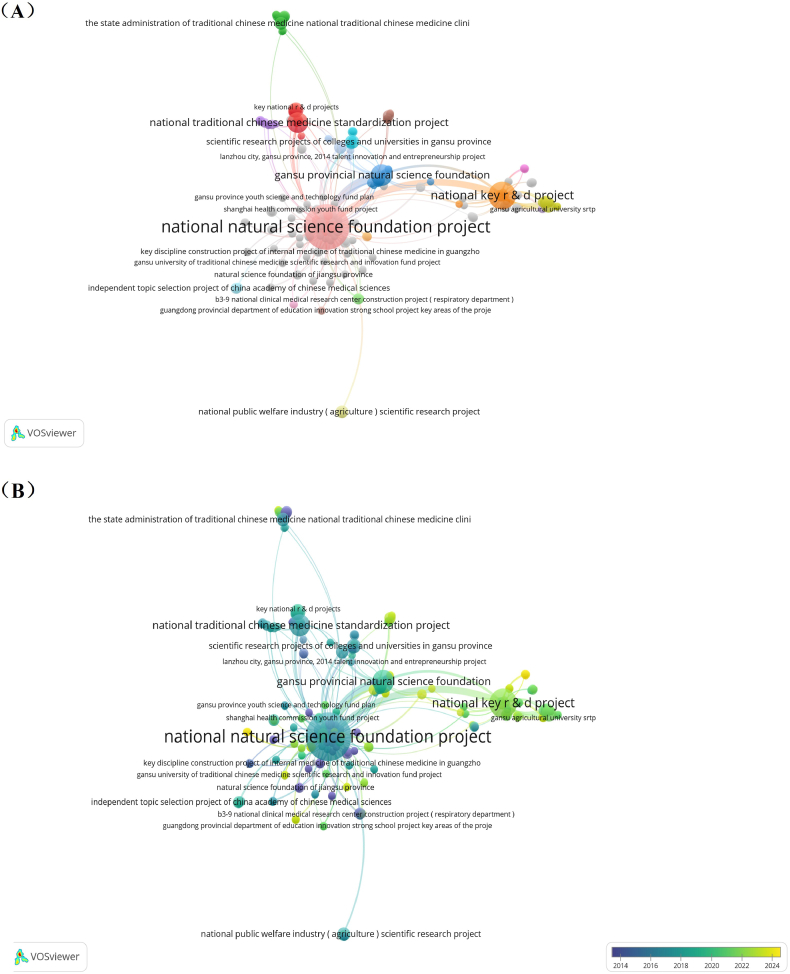
(A) Network map of funding agencies by cluster. (B) Overlay map of network and time of funding agencies.

From Fig. 9 (A), the research on *C. pilosula* was mainly supported by the National Natural Science Foundation of China, at least 116 publications were supported among all output. Then was the major projects of the Ministry of Science and Technology of China, which produced 31 publications. And then was Gansu Provincial Natural Science Foundation produced 17 publications. The National Traditional Chinese Medicine Standardization Project produced 15 publications. The rest of the project produced less than 10 papers. It can be seen that the National Natural Science Foundation of China and the Ministry of Science and Technology of the People 's Republic of China were the core funding agencies for the research of *C. pilosula*. Gansu Province as the main producing area of *C. pilosula* with most productive research scholars set most strongest fund support in China. Moreover, a few other government agencies in Gansu have also set up several similar funds to study *C. pilosula*. In addition, Shaanxi Province and other provinces set small amount of funding, but the degree of autonomy was significantly less than that of Gansu Province. Other departments belongs to national level were also involved in the funding. This shows that as a bulk medicine of traditional Chinese medicine, the research on *C. pilosula* is still valued by local government of the country. The development of *C. pilosula* industry is inseparable from the strong support of the state and local governments. Fig. 9 (B) shows that the Ministry of Science and Technology of the People 's Republic of China has begun support for *C. pilosula*'s research around 2022, and some government agencies in Gansu are still continuing to give fund. Therefore, in the future, funding may still be mainly obtained from national and local government of Gansu, this implies the research of *C. pilosula* has been paid high attention by the national and local government, and the development trend of *C. pilosula*'s industry has a prospecting development in the future.

## 4 Limitations

We acknowledge that our study has several limitations. The data in this paper retrieved publications only from a single database of Web of Science, the publications from other databases, such as PubMed, Embase, Cochrane Library, etc. were not included. Thus, it is possible that some articles worthwhile need to be collected but not included in this database. This may have led to exclusion of some articles [11,13,16]. The funding information retrieved from funding acknowledgment were not complete, since a substantial number of documents only report funding agency or grant number information in respective fields, and articles written in Chinese have a higher funding presence rate than other non-English WoS publications [26,27]. Therefore, researchers should keep abreast of the latest published achievements. However, we consider the Web of Science database as a reliable service for publications and citations, although it may include fewer documents and journals than that of other databases such as Google Scholar or Scopus, so this research provides a solid global perspective on *C. pilosula*'s related research over the past decade and highlights the future research direction.

## 5 Conclusions

In conclusion, this review explored the research history, research frontier, and developing trends in the field of *C. pilosula* research from 1979 to 2024 using bibliometric methods. The results showed research on *C. pilosula* obtained worldwide attention with valuable scientific significance in the past and near future, especially in traditional Chinese medicine. The development of *C. pilosula* underwent through 3 important stages: initial stage (1979–2002), fast development stage (2003–2012), and explosive development stage (2013-present) with rapid growth as indicated by the linear relationship with very good fit at each stage. Considerable attention has been attracted from 1292 institutions across 27 countries of *C. pilosula*, with China played a leading role in publications and collaboration. Lanzhou University has made substantial contributions to research output. Fangdi Hu from Lanzhou University is regarded as a key author and core specialist who has organized researchers in agricultural and pharmacological field, exerting significant influence in the field. The research in *C. pilosula* has established solid cooperation network with fixed institutions of productive specialist in agricultural, medical and chemical field. The research of *C. pilosula* are mostly studied on pharmacological activity, chemical constituents, quality control, polysaccharide and medication rule for network pharmacology study. The research focus on *C. pilosula* has evolved over time to incorporate new scientific frontiers. The research on medication rules, data mining, molecular docking, traditional Chinese medicine, gut microbiota, and polysaccharide are frontier and hot focus both at present and are likely to continue in the near future. The attention and support extended by the national and local government of Gansu province underscore the significance of research on *C. pilosula*. Future research directions are are expected to prioritize traditional Chinese medicine and pharmacological activities, indicating a promising trajectory for *C. pilosula* research. The field is anticipated to advance rapidly, becoming more robust and forward-looking, and continued national and local support is expected to granted continually in the near future for its research and development.

## CRediT authorship contribution statement

**Doudou Yang:** Writing – original draft, Resources, Formal analysis, Data curation, Conceptualization. **Qingqing Zhang:** Data curation. **Zhuodi Wu:** Investigation. **Yuan Chen:** Writing – review & editing. **Ziping Cai:** Formal analysis. **Liang Zhao:** Methodology. **Yinku Liang:** Writing – review & editing, Project administration, Funding acquisition.

## Data availability statement

The original contributions presented in the study are included in the article, further inquiries can be directed to the corresponding author.

## Declaration of competing interest

The authors declare that they have no known competing financial interests or personal relationships that could have appeared to influence the work reported in this paper.

## References

[1] Yang L., Sibbritt D. (2022). Factors associated with Chinese herbal medicine use among middle-aged and older women with arthritis: evidence from China. Sci Rep-Uk.

[2] Fan J., Gao Y., Zhao N. (2020). Bibliometric analysis on COVID-19: a comparison of research between English and Chinese studies. Front. Public Health.

[3] Yang D., Chen Y., Guo F. (2019). Comparative analysis of chemical composition, antioxidant and antimicrobial activities of leaves, leaf tea and root from Codonopsis pilosula. Ind. Crop. Prod..

[4] Gao S., Liu J., Wang M. (2018). Traditional uses, phytochemistry, pharmacology and toxicology of Codonopsis: a review. J. Ethnopharmacol..

[5] Lin J., Shi M., Jin L. (2021). Medication rule of traditional Chinese medicine in treatment of hematological diseases based on data mining. Chin. Tradit. Herb. Drugs.

[6] Youn B.Y., Song H.J., Yang K. (2021). Bibliometric analysis of integrative medicine studies from 2000 to 2019. Am. J. Chin. Med..

[7] Jia X.-Y., Liu Y.-M., Wang Y.-F. (2022). Bibliometric study of soluble guanylate cyclase stimulators in cardiovascular research based on web of science from 1992 to 2021. Front. Pharmacol..

[8] Rocchi L., Boggia A., Paolotti L. (2020). Sustainable agricultural systems: a bibliometrics analysis of ecological modernization approach. Sustainability-Basel.

[9] Yang X., Yin H., Zhang D. (2022). Bibliometric analysis of Cathepsin B research from 2011 to 2021. Front Med-Lausanne.

[10] Kokol P., Blažun Vošner H., Završnik J. (2021). Application of bibliometrics in medicine: a historical bibliometrics analysis. Health Info Libr J.

[11] Lu L., Liu G., Xu Y. (2024). A systematic review of studies on stress during the COVID-19 pandemic by visualizing their structure through COOC, VOS viewer, and Cite Space software. Front. Psychiatr..

[12] Sabe M., Chen C., Perez N. (2023). Thirty years of research on negative symptoms of schizophrenia: a scientometric analysis of hotspots, bursts, and research trends. Neurosci. Biobehav. Rev..

[13] Van Eck N.J., Waltman L. (2017). Citation-based clustering of publications using CitNet Explorer and VOSviewer. Scientometrics.

[14] Li H., Ang Y., Yang W. (2024). Green roof development knowledge map: a review of visual analysis using CiteSpace and VOSviewer. Heliyon.

[15] Visser M., Van Eck N.J., Waltman L. (2021). Large-scale comparison of bibliographic data sources: Scopus, web of science, dimensions, crossref, and microsoft academic. Quantitative science studies.

[16] Liu W., Ni R., Hu G. (2024). Web of science core collection's coverage expansion: the forgotten arts & humanities citation Index?. Scientometrics.

[17] Jin B., Wang B. (1999). Chinese science citation database: its construction and application. Scientometrics.

[18] Leydesdorff L., Bihui J. (2005). Mapping the Chinese Science Citation Database in terms of aggregated journal–journal citation relations. J Am Soc Inf Sci Tec.

[19] Liu W. (2019). The data source of this study is Web of Science Core Collection? Not enough. Scientometrics.

[20] Pal M., Bharati P., Pal M. (2019). Applications of Regression Techniques.

[21] Xiaojin M., Juan G., Jinfu T. (2015). Status and future of natural resource for Chinese materia medica. China J. Chin. Mater. Med..

[22] Liu W. (2021). Caveats for the use of Web of Science Core Collection in old literature retrieval and historical bibliometric analysis. Technol. Forecast. Soc. Change.

[23] Yan Y., Chengwang T. (2016). Key problems in development of modern Chinese medicine. Chin. Tradit. Herb. Drugs.

[24] Hou J., Zheng B., Wang D. (2023). How boundary-spanning paper sparkles citation: from citation count to citation network. Journal of Informetrics.

[25] Ampese L.C., Sganzerla W.G., Di Domenico Ziero H. (2022). Research progress, trends, and updates on anaerobic digestion technology: a bibliometric analysis. J. Clean. Prod..

[26] Liu W., Tang L., Hu G. (2020). Funding information in Web of Science: an updated overview. Scientometrics.

[27] Kokol P., Vošner H.B. (2018). Discrepancies among Scopus, Web of Science, and PubMed coverage of funding information in medical journal articles. J. Med. Libr. Assoc.: JMLA.

